# Treatment patterns for metastatic colorectal cancer in Spain

**DOI:** 10.1007/s12094-019-02279-5

**Published:** 2020-01-23

**Authors:** E. Aranda, E. Polo, C. Camps, A. Carrato, E. Díaz-Rubio, V. Guillem, R. López, A. Antón

**Affiliations:** 1 Oncology Department, Maimonides Institute of Biomedical Research (IMIBIC), Reina Sofía Hospital, University of Córdoba, Córdoba, Spain; 2 CIBERONC, Instituto de Salud Carlos III, Madrid, Spain; 3 Medical Oncology Department, Miguel Servet University Hospital, IIS Aragón, Zaragoza, Spain; 4 Molecular Oncology Laboratory, Fundación Investigación Hospital General Universitario de Valencia, Valencia, Spain; 5 CIBERONC, Valencia, Spain; 6 Department of Medical Oncology, Consorcio Hospital General Universitario de Valencia, Valencia, Spain; 7 Department of Medicine, Universitat de Valencia, Valencia, Spain; 8 Medical Oncology, Ramón y Cajal Universtity Hospital, IRYCIS, Alcalá University, Madrid, Spain; 9 Medical Oncology, Hospital Clínico San Carlos, Madrid, Spain; 10 Medical Oncology Department, Fundación Instituto Valenciano de Oncología, Valencia, Spain; 11 Servicio de Oncología Médica y Grupo de Oncología Médica Traslacional (Oncomet), Hospital Clínico Universitario e Instituto de Investigación Sanitaria (IDIS) de Santiago, Santiago de Compostela, Spain; 12 Oncology Dapartment, Hospital Universitario Reina Sofía, Av. Menendez Pidal, s/n, 14004 Córdoba, Spain

**Keywords:** Colorectal cancer, Metastatic, Treatment patterns, KRAS/BRAF mutation status, Clinical practice guideline

## Abstract

**Purpose:**

The primary aim of this retrospective study was to describe the treatment patterns according to the type of treatment received by patients with metastatic colorectal cancer (mCRC) in Spain.

**Methods:**

This was a retrospective, observational, multicenter study performed by 33 sites throughout Spain that included consecutive patients aged 18 years or older who had received or were receiving treatment for mCRC.

**Results:**

At the time of inclusion, of the 873 evaluable patients, 507 (58%) had received two lines, 235 (27%) had received three lines, 106 (12%) had received four lines, and the remaining patients had received up to ten lines. The most frequent chemotherapy schemes were the FOLFOX or CAPOX regimens (66%) for first-line treatment, FOLFOX, CAPOX or FOLFIRI (70%) for second-line treatment, and FOLFOX, FOLFIRI or other fluoropyrimidine-based regimens for third- and fourth-line (over 60%) treatment. Sixty percent of patients received targeted therapy as part of their first-line treatment, and this proportion increased up to approximately 70% of patients as part of the second-line of treatment. A relevant proportion of patients were treated with unknown KRAS, and especially the BRAF, mutation statuses.

**Conclusions:**

This study reveals inconsistencies regarding adherence to the recommendations of the ESMO guidelines for the management of mCRC in Spain. Improved adherence to the standard practice described in such guidelines for the determination of RAS and BRAF mutation statuses and the use of targeted therapies in first-line treatment should be considered to guarantee that patients can benefit from the best therapeutic approaches available.

## Introduction

Colorectal cancer (CRC) is a leading cause of cancer-related morbidity and mortality worldwide, and its global burden is expected to increase in the coming years [1]. By the time of diagnosis, approximately 25% of patients exhibit metastatic disease, and almost 50% of patients will develop metastasis during the course of the disease [2]. Overall, the clinical outcome of metastatic (m) CRC has improved in the last decade, probably as a consequence of the availability of improved systemic treatment options and the improvements in the management of the disease [3]. Management of mCRC should be guided by the available clinical practice guidelines (CPGs) [3, 4]. However, treatment patterns in patients with mCRC may vary from country to country [5] and even within the same country due to variations in practice and/or restrictions for the use of some drugs [6, 7]. In addition, adherence to the guidelines for managing CRC may also vary and has been reported as acceptable in some countries [6, 8] and suboptimal in others [7]. In a recent study conducted in the Netherlands, the treatment strategies agreed with the national guidelines in only two-thirds of the patients with stage II colon cancer, and targeted therapy was not routinely administered as first-line treatment in a substantial proportion of patients with mCRC [7]. In addition to the factors mentioned above, it has been reported that age, sex, ethnicity, type of residence and geographical region may affect access to KRAS mutation testing and that these disparities in access to mutation analysis could be responsible, at least in part, for a reduced adherence to the guidelines on this regard [9, 10]. Incomplete or unreliable tests in some laboratories could also affect adherence to biomarker testing guidelines [11].

Adherence to CPGs is of paramount interest because CPGs have the potential to reduce inappropriate practice variations, improve the translation of research evidence into clinical practice and improve the quality and safety of healthcare [12]. Adherence to CPGs has been associated with better treatment outcomes in patients with breast cancer [13], cervical cancer [14] and colorectal cancer [15, 16]. Information on treatment patterns for mCRC in Spain is limited to a study conducted with a private database (LifeLink Oncology Analyzer Database) in France, Germany, Italy and Spain that included 649 patients from Spain, who were mostly patients of medical oncologists [5], and a single-center study that included 157 patients [17]. These studies provide some information on how mCRC is treated in Spain and different treatment patterns from those in other European countries, which could be explained by differences in local treatment guidelines, physician prescribing behaviors or reimbursement policies [5]; additionally, in accordance with other European studies, many laboratories were not ready for biomarker testing in the context of anti-EGFR therapy [17]. However, these studies did not evaluate whether the treatment patterns were consistent with the recommendations of the CPGs.

The primary aim of this retrospective study was to describe the treatment patterns according to the type of treatment received by patients with mCRC in Spain. The secondary objectives were to describe these patterns by ECOG performance status and *KRAS* mutation status and to describe the adherence of those treatment patterns to the ESMO and SEOM guidelines for the treatment of mCRC.

## Patients and methods

The STREAM (Study on the TREAtment of Metastatic colorectal cancer) study was a retrospective, observational, multicenter study performed by 33 sites throughout Spain. The study was approved by a clinical research ethics committee. After being informed about the study, all patients gave their written informed consent before any study procedures were performed.

The study included consecutive patients aged 18 years or older who had received or were receiving treatment for mCRC before being enrolled in the study and who provided their written informed consent. Patients were excluded if they exhibited a cognitive impairment that precluded their understanding of the study characteristics, as they were described in the patient information sheet. The inclusion of patients participating in clinical trials was allowed.

The following information was collected by the investigators from the patients’ medical histories between the date of diagnosis and the day prior to signing the informed consent form: demographic and anthropometric data; data related to the initial diagnosis, including date, primary tumor location and clinical stage based on the TNM classification; diagnosis of metastatic disease, including date of diagnosis, location of metastases and clinical stage as per the TNM classification; KRAS/BRAF mutation status; ECOG performance status; and treatment for metastatic disease, including treatment line and scheme, start and end dates, reason for treatment discontinuation, best response and date of progression for each of the treatment lines received.

Based on data from a large cohort of patients with mCRC, it was estimated that 28% of patients with mCRC receive at least three lines of treatment [18], and thus this subgroup of patients could reflect the most frequent treatment patterns. To obtain a proportion of patients with a precision of ± 3% and a 95% confidence interval, a total of 844 patients were required; assuming that 5% of patients had missing data, it was calculated that a total of 889 patients were required.

Continuous outcomes were described with the mean and standard deviation or with the median and interquartile range when required. Categorical outcomes were described using the absolute and relative frequencies. To evaluate adherence to the clinical practice guidelines, we focused on the ESMO guidelines issued in 2012 [19], 2014 [20] and 2016 [3]. The ranges of dates for each of these evaluations were 30-06-2010 to 04-09-2014 for adherence to ESMO 2012, 04-09-2014 to 08-07-2016 for adherence to ESMO 2014, and after 08-07-2016 for adherence to ESMO 2016. A specific guideline was considered applicable to a particular patient if the date of the diagnosis was within the above-mentioned range of dates. We considered that treatment that was not supported by the corresponding guidelines (e.g., gemcitabine) as non-adherent to those regimens. Regarding *KRAS/NRAS/BRAF* mutation status, we considered that the patient was non-adherent with all three ESMO guidelines if the *KRAS* mutation status was unknown; non-adherent with the 2014 and 2016 ESMO guidelines if the *NRAS* mutation status was unknown in patients with *KRAS*wt; and non-adherent with the 2016 ESMO guidelines if the *BRAF* mutation status was unknown in patients *RAS*wt. All analyses were performed using IBM SPSS version 22 (IBM, Armonk, New York, United States).

## Results

### Patient disposition and demographic and clinical characteristics

Thirty-three sites recruited 936 patients between November 2016 and April 2017. Of these patients, we excluded 63 patients, mainly because they had several unresolved queries, thus leading to 873 patients who were included in the final analyses.

The patients had a median (interquartile range) age of 66.0 (59.0–73.0) years, were predominantly women (64%) and had ECOG performance status of 0 or 1 (94%). The primary tumor was most frequently located in the left-sided colon (68%), and the most frequent metastatic site was the liver (70%). The baseline characteristics are shown in Table 1.

**Table 1:** Patient and tumor characteristics

Characteristic	*N*	
Age,	873	
Median (IQR)		66.0 (59.0–73.0)
> 75 years, *n* (%)		133 (15.2)
Sex (male), *n* (%)	873	556 (63.7)
Body mass index, mean (SD)	750	26.5 (4.5)
Time from mCRC diagnosis to inclusion in the study (months), median (IQR)	873	16.5 (7.6–30.9)
ECOG performance status, (%)	847	
0		387 (45.7)
1		410 (48.4)
≥ 2		50 (5.9)
Colorectal tumor location, *n* (%)	873	
Left		595 (68.2)
Right		227 (26.0)
Multiple		7 (0.8)
Unknown		44 (5.0)
Metastatic sites ≥ 3, *n* (%)	873	80 (9.2)
Metastatic sites, *n* (%)	873	
Liver		610 (69.9)
Lung		286 (32.8)
Peritoneum		158 (18.1)
Distant lymph nodes		116 (13.3)
Others		94 (10.8)
RAS status, *n* (%)	873	
Unknown		104 (11.9)
Mutated		374 (42.8)
Wild-type		395 (45.2)
KRAS status, *n* (%)	374	
Unknown		5 (1.3)
Mutated		349 (93.3)
Wild-type		5 (1.3)
NRAS status, *n* (%)	374	
Unknown		203 (54.3)
Mutated		65 (17.4)
Wild-type		19 (5.1)
BRAF status, *n* (%)	873	
Unknown		638 (73.1)
Mutated		20 (2.3)
Wild-type		215 (24.6)

*ECOG* Eastern Cooperative Oncology Group, *IQR* interquartile range, *n* number of patients with the parameter, *N* number of evaluable patients for the parameter, *SD* standard deviation

### Pattern of treatment with chemotherapy and targeted therapies

**Fig. 1:**
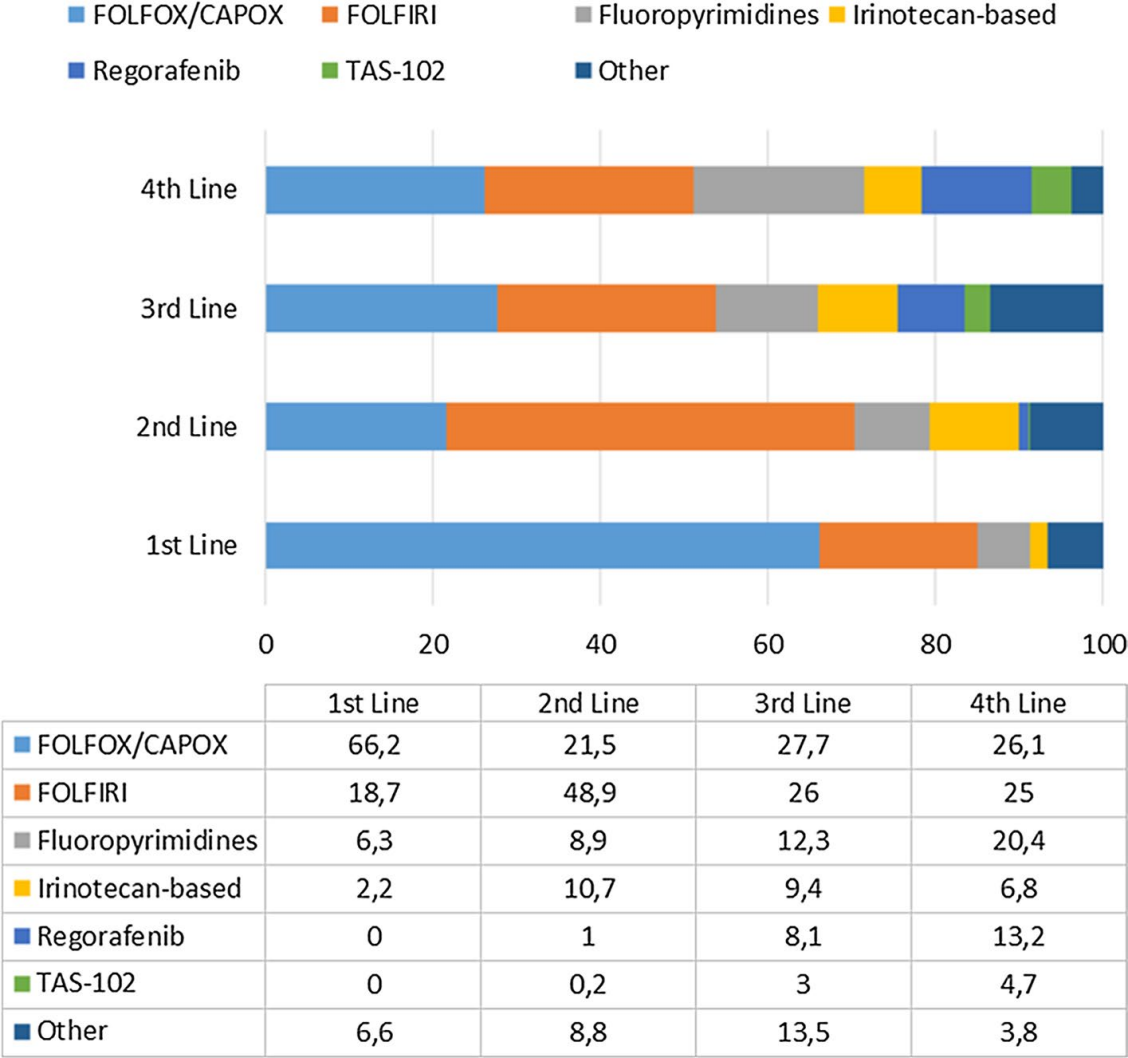
Treatment schemes from first-line to fourth-line chemotherapy in patients with metastatic colorectal cancer in Spain. *CAPOX* capecitabine/oxaliplatin, *FOLFIRI* 5-FU/leucovorin/irinotecan, *FOLFOX* 5-FU/leucovorin/oxaliplatin, *TAS-102* Trifluridine/tipiracil

Overall, at the time of inclusion, 873 patients had received one line of treatment, 507 (58%) had received two lines, 235 (27%) had received three lines, 106 (12%) had received four lines, and the remaining patients had received up to ten lines. The most frequent chemotherapy schemes were the FOLFOX or CAPOX regimens (66%) for first-line treatment, FOLFOX, CAPOX or FOLFIRI (70%) for second-line treatment, and FOLFOX, FOLFIRI or other fluoropyrimidine-based regimens for third- and fourth-line (over 60%) treatment (Fig. 1). The most frequent specific regimens for the first four lines of chemotherapy are presented in Table 2. In patients aged 75 years or older, the most frequent regimens were as follows: capecitabine (17%), CAPOX (16%), and FOLFOX (12%) as first-line treatment; irinotecan (13%), capecitabine (10%) and FOLFIRI (10%) as second-line treatment; FOLFOX (18%), FOLFIRI (8%), irinotecan + cetuximab (8%), capecitabine (8%) and panitumumab (8%) as third-line treatment; and capecitabine (32%), FOLFOX (16%) and regorafenib (16%) as fourth-line treatment.

**Table 2:** Most frequent treatment regimens from first-line to fourth-line chemotherapy in patients with metastatic colorectal cancer in Spain

Line	Regimen	*n*	%
First *N* = 873	FOLFOX-Bevacizumab	156	17.9
	FOLFOX	113	12.9
	CAPOX	101	11.6
	CAPOX-Bevacizumab	68	7.8
	FOLFOX-Panitumumab	67	7.7
	FOLFIRI-Bevacizumab	51	5.8
	FOLFIRI	47	5.4
Second *N* = 507	FOLFIRI-Bevacizumab	70	13.8
	FOLFIRI	59	11.6
	FOLFIRI-Aflibercept	57	11.2
	FOLFOX-Bevacizumab	35	6.9
	FOLFIRI-Cetuximab	31	6.1
	FOLFIRI-Panitumumab	29	5.7
	FOLFOX	29	5.7
Third *N* = 255	FOLFOX	26	11.1
	FOLFIRI	19	8.1
	Regorafenib	19	8.1
	FOLFOX-Bevacizumab	18	7.7
	Capecitabine-Bevacizumab	13	5.5
	FOLFIRI-Bevacizumab	13	5.5
	FOLFIRI-Cetuximab	13	5.5
Fourth *N* = 106	Regorafenib	14	13.2
	FOLFIRI-Bevacizumab	9	8.5
	Capecitabine	8	7.5
	FOLFOX	7	6.6
	Capecitabine-Bevacizumab	6	5.7
	FOLFIRI	6	5.7
	FOLFIRI-Cetuximab	6	5.7
	FOLFOX-Bevacizumab	6	5.7

*CAPOX* capecitabine/oxaliplatin, *FOLFIRI* 5-FU/leucovorin/irinotecan, *FOLFOX* 5-FU/leucovorin/oxaliplatin, *n* number of patients with the characteristic

**Fig. 2:**
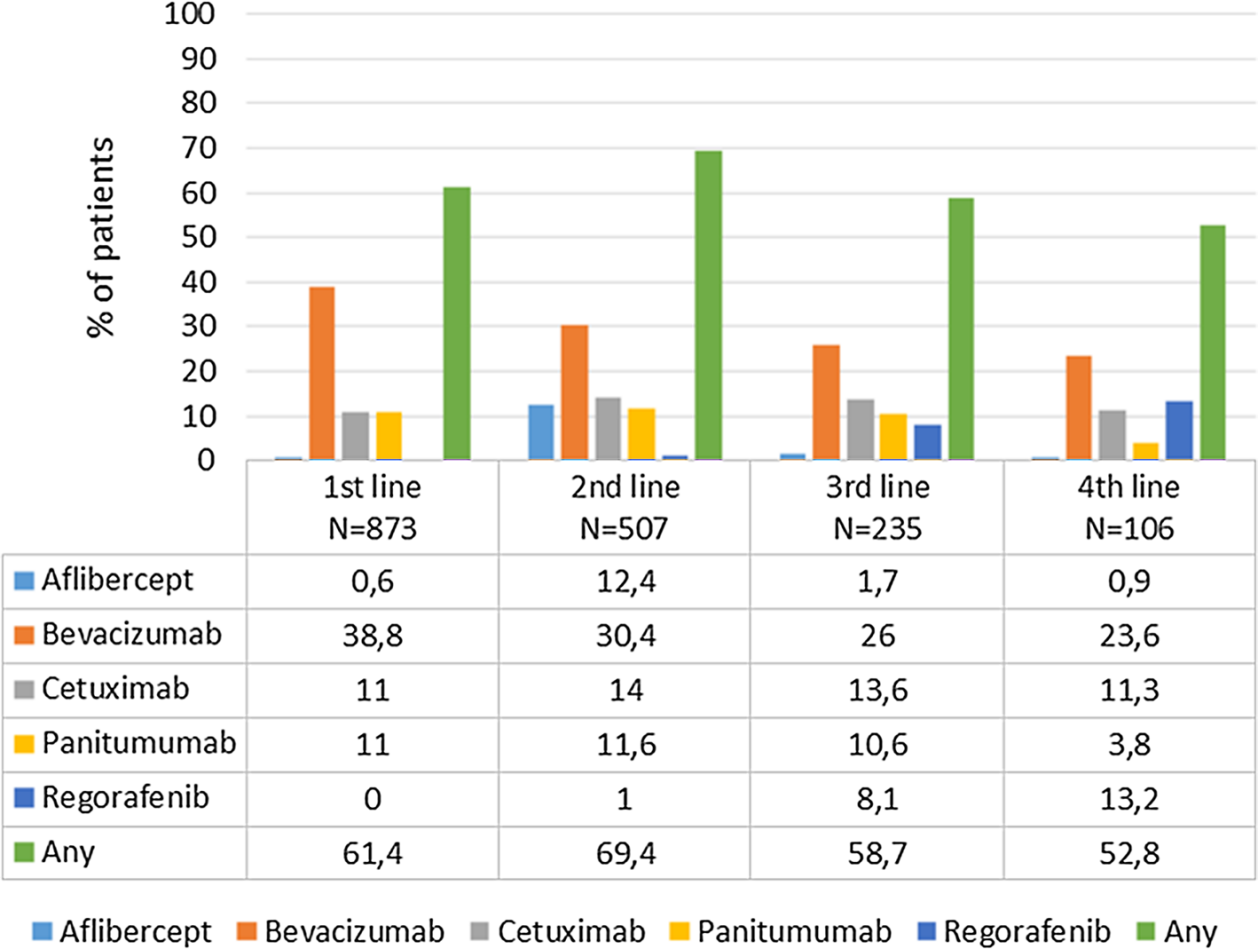
Use of targeted therapies by treatment line in patients with metastatic colorectal cancer in Spain

Sixty percent of patients received targeted therapy as part of their first-line treatment, and this proportion increased up to approximately 70% of patients as part of the second line of treatment (Fig. 2). The most frequent targeted therapy, regardless of the line of treatment, was bevacizumab, which was prescribed in almost 40% of the patients as first-line treatment, and this proportion decreased thereafter to 24% as fourth-line treatment. Regorafenib was almost not used until the third or fourth line, where it was used in approximately 10% of patients.

### Adherence to ESMO guidelines

The *KRAS* mutation status was unknown in 10–12% of the patients according to the ESMO guidelines from 2012, 2014 and 2016, the *NRAS* mutation status was known in almost 100% of the patients with *KRASwt* according to the ESMO guidelines from 2014 and 2016, and the *BRAF* mutation status was unknown in half of the patients who were *RASwt* according to the ESMO guidelines from 2016 (Table 3). Between 3 and 14% of the patients received a chemotherapy regimen in the first three lines of treatment that was not supported by the corresponding ESMO guidelines (Table 3).

**Table 3:** Adherence to the European Society of Medical Oncology guidelines in patients with metastatic colorectal cancer in Spain

Variable	ESMO 2012 *N* = 146	ESMO 2014 *N *= 430	ESMO 2016 *N* = 229
Unknown *KRAS* mutation status, *n* (%)	18 (12.3)	43 (10.0)	28 (12.2)
Unknown *NRAS* mutation status among those with *KRASwt* n/N (%)		1/199 (0.5)	0/98 (0.0)
Unknown *BRAF* mutation status among those with *RASwt*, n/N (%)	–		44/90 (48.9%)
Chemotherapy regimens not supported by the guideline, *n* (%)			
First	11 (7.5)	27 (6.3)	17 (7.4)
Second	6 (12.8)	11 (8.8)	1 (3.3)
Third	5 (14.3)	5 (13.9)	–
Fourth	3 (20.0)	3 (33.3)	–
Other issues		− 38 (8.8%) patients receiving second-line therapy did not switch to an irinotecan- or oxaliplatin-containing combination regimen according to the guidelines	− 10 (4.4%) patients receiving second-line therapy did not switch to an irinotecan- or oxaliplatin-containing combination regimen according to the guidelines − 46 of the 192 (24.0%) patients who received treatment with anti-EGFR in the first line and 22 of the 130 (16.9%) patients who received treatment with anti-EGFR in the second line received the same treatment again in a later line, despite the fact that the guidelines do not recommend a retreat

## Discussion

This study, which was conducted in a fairly representative sample of patients with mCRC treated in Spain, shows that the most frequent chemotherapy regimens for treating this condition are FOLFOX, CAPOX, and FOLFIRI, in accordance with the ESMO clinical practice guidelines for the management of mCRC [3]. In addition, this study shows that the use of targeted therapies for the treatment of metastatic disease is not fully consistent with the recommendations of the CPGs. In addition, a relevant proportion of patients were treated with unknown *KRAS*, and especially the *BRAF*, mutation statuses.

The demographic and clinical characteristics of our sample are similar to those of a previous multicenter, multinational study that also included a sample of 649 patients in a study conducted in 2009 in Spain [5]. This study used physician sampling frames based on the distribution of oncology-treating providers in each country [5] and therefore, the cohort was likely to be representative of the patients of the participating country. Thus, this suggests that our sample is fairly representative of the patients with mCRC treated in Spain.

There was a substantial degree of overlap among the most frequent chemotherapy regimens (namely, FOLFOX, CAPOX and FOLFIRI) administered as first- and second-line treatment for metastatic disease, and to a lesser extent, with those administered as third-line treatment. This result suggests that an important proportion of patients are retreated with those regimens. Overall, the use of targeted therapies was lower than expected, especially in first-line treatment; despite being recommended by clinical practice guidelines [3], only 60% of the patients were treated with a targeted therapy. This is an important issue since targeted therapies have demonstrated a significant survival benefit when administered in combination with chemotherapy during first-line and second-line therapy for patients with mCRC [21]. This issue is further complicated by the fact that in our sample, a substantial proportion of the patients were treated without a known RAS mutation status, and anti-epidermal growth factor receptor therapies should be restricted to patients with a *RAS*wt status. Therefore, delaying the introduction of targeted therapy is a suboptimal practice according to the CPGs. The difficulties in accessing some oncologic drugs in several Spanish regions, as reflected by SEOM [22], could impact the adherence to the recommendations of the CPGs. An analysis by region could clarify this issue, but unfortunately, our sample was not large enough for such an analysis.

Regarding the adherence to CPGs by the employed regimens, except for the abovementioned situation with the use of targeted therapies, the majority of chemotherapy regimens were among those recommended in the CPGs. However, the situation with the determination of the *RAS* and *BRAF* mutation status requires consideration. The benefit of determining the RAS and BRAF mutation status for the management of mCRC is well known [23] since it allows personalization of the treatment approach; similarly, the 2016 edition of the ESMO clinical practice guidelines recommend that “RAS testing should be carried out on all patients at the time of diagnosis” and that the *BRAF* mutation status “should be assessed alongside the assessment of tumor *RAS* mutational status for prognostic assessment” [3]. In our study, among the patients diagnosed after July 2016, 12% of these patients had an unknown *KRAS* mutation statuses and, of those with wild-type *RAS*, almost 50% of the *BRAF* mutation statuses were unknown. This finding deserves further research to elucidate the barriers that preclude the determination of the *RAS* mutation status*,* and especially the *BRAF* mutation status.

The main limitations of the study are its retrospective nature and the use of a nonprobabilistic convenience sample. The former limits the quality of the information recorded, and the latter limits the generalizability of the study; the inclusion of consecutive patients limits the potential for selection bias, to a certain extent. In addition, since written informed consent was required, patients with more advanced disease could potentially be less represented in our sample; thus, approximately half of the patients had ECOG performance status of 0. In addition, due to the limited information recorded, we only evaluated gross markers of adherence to the treatment guidelines.

In conclusion, this study reveals inconsistencies regarding adherence to the recommendations of the ESMO guidelines for the management of mCRC in Spain. Improved adherence to the standard practice described in such guidelines for the determination of RAS and BRAF mutation statuses and the use of targeted therapies in first-line treatment should be considered to guarantee that patients can benefit from the best therapeutic approaches available.
